# Dataset of microbial community structure in alcohol sprayed banana associated with ripening process

**DOI:** 10.1016/j.dib.2020.105216

**Published:** 2020-02-05

**Authors:** Fenny Martha Dwivany, Fidya Syam, Husna Nugrahapraja, Ocky Karna Radjasa, Maelita Ramdani Moeis, Susumu Uchiyama

**Affiliations:** a School of Life Sciences and Technology, Institut Teknologi Bandung, Bandung 40132, Indonesia; b Biosciences and Biotechnology Research Center, Institut Teknologi Bandung, Bandung 40132, Indonesia; c Bali International Research Center for Banana, Badung, Bali 80361, Indonesia; d Department of Marine Science, Faculty of Fishery and Marine Science, Diponegoro University, Semarang 50275, Central Java, Indonesia; e INABIG Research Institute, Bandung 40135, Indonesia; f Department of Biotechnology, Graduate School of Engineering, Osaka University, 2-1 Yamadaoka, Suita, Osaka 565-0871, Japan

**Keywords:** Amplicon sequencing, *Musa acuminata* AAA group, Endophytic, Epiphytic, *Alcaligenes*

## Abstract

Banana ripening is a complex molecular process that produces visible changes in the texture, aroma, taste and nutritional content. Ripening is controlled by genetic code, metabolic pathway and associated microbiome. We reported the microbial community structure during banana ripening with alcohol treatment to discover endophytic and epiphytic microbes. We observed the pulp and peel from the first and seventh days of Cavendish (*Musa acuminata* cv. Cavendish) from mature green fruit and treated with 70% alcohol or distilled water sum up to eight samples and applied the 16S rRNA Illumina sequencing from V3–V4 gene region. After quality check 144,368 sequences were obtained in the dataset comprising a total read length of 1,237,805 base pairs. A sum of 199 genera were successfully isolated, with genera *Alcaligenes* was the most dominant genera at 56.65% and followed by more than 1% were genera *Acinetobacter*, *Enhydrobacter*, *Pseudomonas*, *Stenotrophomas*, *Thermus*, and *Aerococcus* using mothur pipelines. The highest diversity sample with 101 unique genera was belongs to distilled water treated raw bananas peel (NN1K) and the lowest diversity at 38 was belongs to distilled water treated ripe bananas pulp (NN7D). The metagenome data are available at NCBI Sequence Read Archive (SRA) database and Biosample under accession number PRJNA590572. The data contribute to discover different bacterial communities during post-harvest treatment.

**Table:** Specification Table

Subject	Biological Sciences
Specific subject area	Fruit ripening microbiome
Type of data	Table Figure Chart Graph 16S rRNA sequences and analysis
How data were acquired	NGS Sequencing on Illumina HiSeq 2500 platform
Data format	Raw Analyzed
Parameters for data collection	Microbial genomics DNA collected from the pulp and peel of Cavendish banana at first and seventh days from mature green fruit and treated using 70% alcohol and distilled water are used as template to amplify the V3–V4 of 16S rRNA gene
Description of data collection	Comparison of microbial communities from the pulp and peel of Cavendish banana at first and seventh days of fruit ripening and treated using 70% alcohol and distilled water
Data source location	The samples were collected from Genetics and Molecular Biotechnology Laboratory, School of Life Sciences and Technology, Institut Teknologi Bandung, Bandung, West Java, Indonesia 40132 (6°53′28.9″S 107°36′38.3″E)
Data accessibility	Data is within this article and all sequences generated in this research are submitted to NCBI SRA under the accession numbers SRS5694892 up to SRS5694899 being available in the NCBI BioSample Submission Portal as Bioproject PRJNA590572 (https://www.ncbi.nlm.nih.gov/bioproject/PRJNA590572/)
Related research article	C. Lustriane, F.M. Dwivany, V. Suendo, M. Reza, Effect of chitosan and chitosan nanoparticles on postharvest quality on banana fruits, J. Plant. Biotechnol. 43 (2018) 36–44.

**Table:** 

**Value of the Data** • These metagenome data provide the first information of microbial structure in response to alcohol spraying during banana ripening. • These metagenome data crucial to identify candidate microbe involved in fruit ripening in response to alcohol spraying. • These metagenome data useful to design a better post-harvest technology using anti-microbial to prolong banana fruit ripening.

## 1 Data description

A metagenome-based approach was used to assess the taxonomic affiliation and function potential of microbial populations in raw and ripe banana's pulp and peel with sterilization using 70% alcohol to identify endophyte microbe and distilled water to identify whole microbe. Total raw reads from all samples before processed was 1,237,805 base pairs. Total number of amplicon sequences reads after quality control, chimera and contaminant removal obtained from ripe banana was 136,479 reads and 7889 reads from raw banana were used in the metagenomic analyses, respectively. Taxonomic analysis yielded a total of 13 classifiable phyla with Proteobacteria was dominant in the entire sample.

**Fig. 1:**
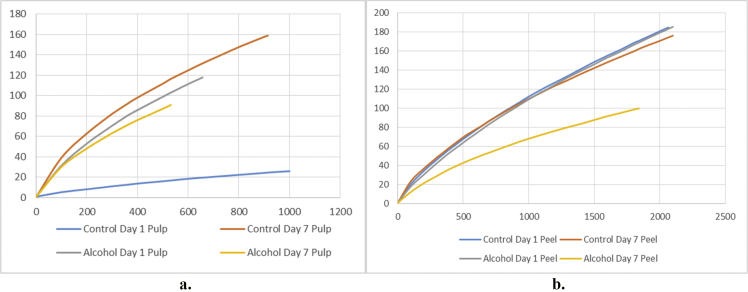
Rarefaction curves a) pulp b) peel sample.

**Fig. 2:**
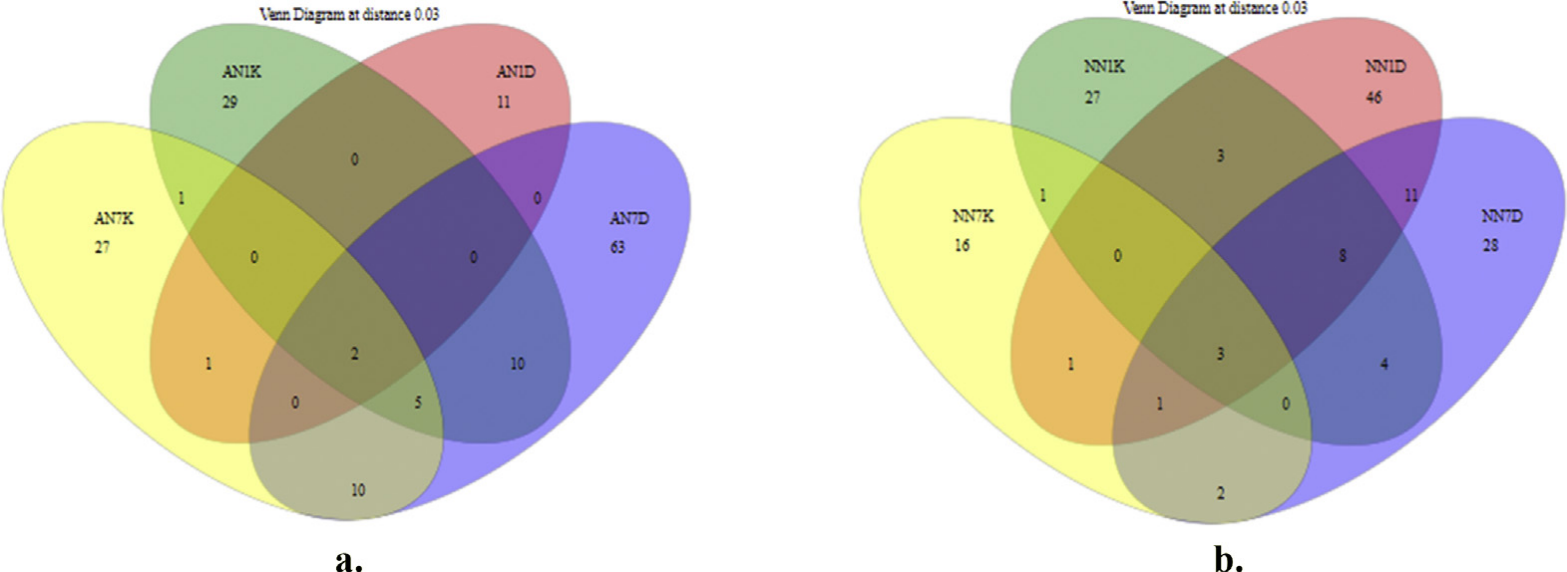
Shared OTU flower diagram a) alcohol b) control sample.

**Fig. 3:**
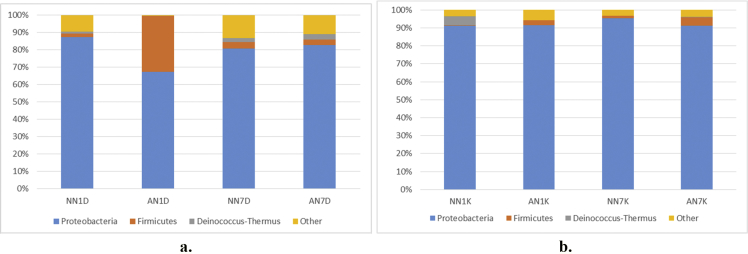
Taxonomic diversity and relative abundance at phyla level a) pulp b) peel sample. Sample code, first and second letter: AN = Alcohol treatment NN = Distilled water treatment; third letter: 1: raw (day 1) banana 7: ripe (day 7) banana, fourth letter: D = banana's pulp K = banana's peel.

**Fig. 4:**
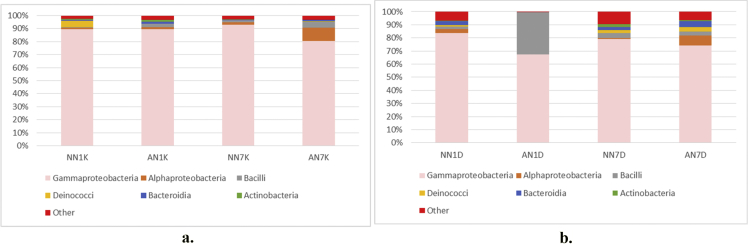
Taxonomic diversity and relative abundance at class level a) pulp b) peel sample. Sample code, first and second letter: AN = Alcohol treatment NN = Distilled water treatment; third letter: 1: raw (day 1) banana 7: ripe (day 7) banana, fourth letter: D = banana's pulp K = banana's peel.

**Fig. 5:**
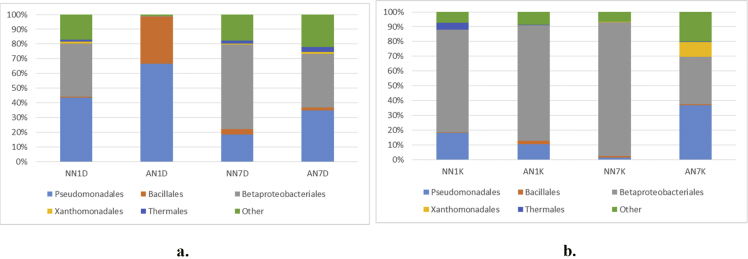
Taxonomic diversity and relative abundance at order level a) pulp b) peel sample. Sample code, first and second letter: AN = Alcohol treatment NN = Distilled water treatment; third letter: 1: raw (day 1) banana 7: ripe (day 7) banana, fourth letter: D = banana's pulp K = banana's peel.

**Fig. 6:**
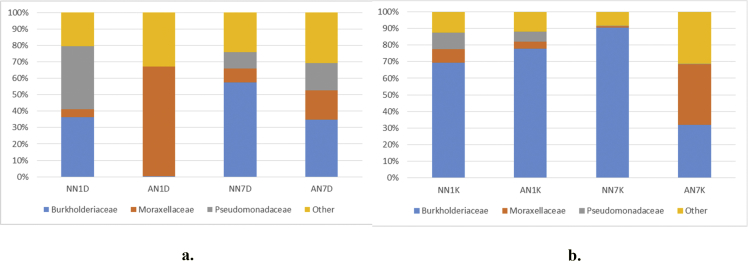
Taxonomic diversity and relative abundance at family level a) pulp b) peel sample. Sample code, first and second letter: AN = Alcohol treatment NN = Distilled water treatment; third letter: 1: raw (day 1) banana 7: ripe (day 7) banana, fourth letter: D = banana's pulp K = banana's peel.

**Fig. 7:**
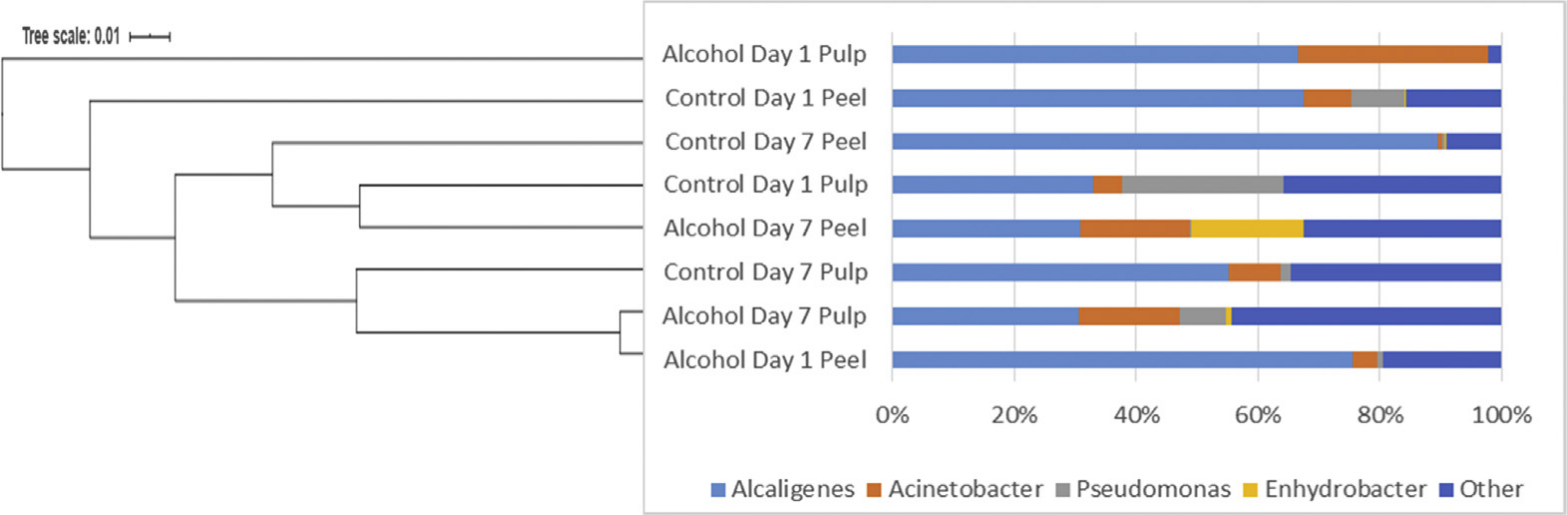
**S**pecies relative abundance and distribution in genera level.

Fig. 1 and Table 1 provide the species diversity by rarefaction curves and the overview of the sequence reads. Fig. 2 shows flower diagram based on shared OTUs distribution for alcohol and control sample, while an UPGMA cluster tree which was based on Jaccard coefficient rich estimator, species relative abundance and distribution in phylum level is shown in Fig. 3. Class level is shown in Fig. 4, order level is shown in Fig. 5, family level in Fig. 6, genera level in Fig. 7.

**Table 1:** Details about the Illumina sequencing metagenome analysis on Cavendish banana during ripening.

Sample _name	Total_reads (PE)	After_ Screening _Read	After _Chimera_ Removal _Reads	After_Chloro plast_and _Mitochondrial _DNA_Removal	Coverage	Sobs	Inverted _simpson
AN1D	157,002	134,740	128,257	128,210	0.99244	1153	1.89437
AN7K	161,216	137,187	135,032	4602	0.96371	281	6.68109
AN1K	142,660	120,829	119,089	2067	0.94001	185	1.84677
AN7D	170,241	145,037	143,551	914	0.90372	159	8.76686
NN1K	152,183	128,226	125,524	5545	0.95635	362	2.24349
NN7K	173,053	147,136	145,796	1841	0.96632	100	1.29589
NN1D	146,956	124,242	122,905	657	0.88736	118	6.10903
NN7D	134,494	113,356	111,443	532	0.89285	91	3.40532

The data are useful for understanding the microbial diversity associated with fruit ripening, and how alcohol treatment may influence the dynamics of microbial communities. Therefore, the data will be useful to design a better postharvest technology using antimicrobial material to prolong banana fruit ripening.

## 2 Experimental design, materials, and methods

### 2.1 Materials

The mature green Cavendish (*Musa acuminata* AAA group) aged at ninth weeks were exposed to ethylene for 24 hours and delivered from PT. Sewu Segar Nusantara, Indonesia. Each banana finger then selected for absence of physical defects on the skin or the pulp and evenness of physiological age, colour and size [1,2].

### 2.2 Sterilization and DNA extraction

Bananas from the first and seventh day were sterilized by alcohol 70% or distilled water (as control). Sterilization was carried out by flowing distilled water or 70% alcohol 3 times throughout the body of banana [3]. Sample for DNA extraction was then prepared by separating peel and pulp of middle part sterilized banana in thick transverse section with width 2–3 cm. The sample was stored in −80 °C freezer. DNA extraction was carried out using the CTAB method [4] from banana's peel and pulp with some modification.

### 2.3 Libraries preparation and amplicons generation

DNA isolates were then used as a template for the construction of 16s rRNA library and NGS Ilumina sequencing with metagenomic analysis approach by Macrogen Korea.

### 2.4 OTU clustering, species annotation, taxon relative abundance and phylogenetic reconstruction

Data sequence were then processed using the mothur v.1.42.0 program [5] with Miseq SOP procedure from the Schloss lab [6]. Analysis was started by merging forward and reverse sequence to make contig with Needleman alignment using minimum Phred score 20 [7]. Then, the data was getting quality control by making sure that sequence length is in around 440–480, having no ambiguous base calls and maximum homopolymer 8. Cleaned data then getting de-replicated into unique sequence and aligned with SILVA 132 database [8] with 1,861,569 rRNA gene sequence SSU bacteria [9]. Aligned sequence was then getting cleaned by removing chimeric sequence with UCHIME program [10] and by removing contaminant (mitochondria and chloroplast). The remaining sequence then getting clustered with OTU similarity 97%. Taxonomic classification from OTU was done 100 times with cutoff value 80 [11]. OTU alignment was done with Wang methods [12] with kmer size 8 based on SILVA 132 database. Phylogenetic tree was visualized with iTOL (Interactive Tree of Life) tool [13].

### 2.5 Diversity analyses and indices

Alpha diversity was calculated with mothur pipeline in order to analyze the complexity of species diversity (Table 1) and species relative abundance and distribution in phylum (Fig. 3), class (Fig. 4), family (Fig. 5), order (Fig. 6), and genera (Fig. 7) level. Rarefaction curves (Fig. 1) were used to estimate coverage and to determine whether a data set is close to saturation [14]. Flower diagram was generated (Fig. 2) according to OTUs clustering. To evaluate the complexity differences between samples in terms of species complexity, beta diversity analysis was employed. An unweighted pair sample UPGMA clustering which made tree based on Jaccard coefficient rich estimator (Fig. 7).
